# Hashimoto’s Thyroiditis: A “Double-Edged Sword” in Thyroid Carcinoma

**DOI:** 10.3389/fendo.2022.801925

**Published:** 2022-02-24

**Authors:** Jiangyue Xu, Ke Ding, Lan Mu, Jiangsheng Huang, Fei Ye, Yu Peng, Can Guo, Chutong Ren

**Affiliations:** ^1^ Department of General Surgery Thyroid Specialty, The Second Xiangya Hospital of Central South University, Changsha, China; ^2^ Department of Urology, Xiangya Hospital, Central South University, Changsha, China

**Keywords:** Hashimoto’s thyroiditis (HT), thyroid carcinoma (TC), meta-analysis, papillary thyroid carcinoma, BRAF^V600E^ mutation

## Abstract

**Background:**

The prevalence of thyroid carcinoma (TC) and Hashimoto’s thyroiditis (HT) has been increasing dramatically over the past decades. We investigated the relationship between HT and TC.

**Methods:**

We followed the Meta-analysis of Observational Studies in Epidemiology (MOOSE) guidelines for carrying out and reporting this meta-analysis. The literature from January 1, 2010 to December 31, 2020, regardless of region and publication type, was searched comprehensively in PubMed, Embase, Web of Science, and Cochrane Library databases. After careful selection and data extraction, the pooled odds ratio of various clinical characteristics in 39 studies were calculated. Publication bias was analyzed using funnel plots.

**Results:**

Meta-analysis of 39 original research articles showed HT to be a risk factor of TC (pooled odds ratio = 1.71; 95% confidence interval, 1.57–1.80; *p <* 0.00001) and papillary thyroid carcinoma (1.67, 1.51–1.85, <0.00001). Patients with papillary thyroid carcinoma (PTC) combined with HT were more likely to have multifocal carcinomas. The prevalence of an extrathyroidal extension, metastasis, BRAF^V600E^ mutation, and recurrence was significantly lower in patients with PTC combined with HT.

**Conclusions:**

HT is a “double-edged sword” in TC patients. HT increases the risk of TC and PTC but is a protective factor against PTC progression.

## 1 Introduction

Thyroid carcinoma (TC) is the most common malignant disease of the endocrine system (1, 2). The overall survival from TC is high, and the prognosis is good, especially for papillary thyroid carcinoma (PTC). However, in recent decades, TC incidence has been increasing dramatically (3). The incidence of autoimmune thyroid diseases, especially Hashimoto’s thyroiditis (HT), has also increased in recent decades (4). About one-third of PTC patients also have HT, and the number of people with PTC combined with HT is increasing (5, 6). The underlying reason for this increased incidence may be improved diagnostic tools (e.g., fine-needle aspiration, high-resolution ultrasound, and detection of thyroid gland-specific antibodies). About 20%–30% of HT patients will eventually experience hypothyroidism, and an increased serum level of thyroid-stimulating hormone (TSH) may promote TC occurrence (5, 6). In addition, exposure to adverse environmental factors can make people more vulnerable to thyroiditis (7, 8).

In 1893, Rudolf Virchow was the first to propose a link between chronic inflammation and cancer development. In the next century, his hypothesis was confirmed in several human diseases (9, 10). The most convincing evidence for his hypothesis was (i) the link between chronic inflammatory diseases of the intestine (Crohn’s disease and ulcerative proctocolitis) and colon adenocarcinoma, (ii) chronic infection with the hepatitis B virus or hepatitis C virus and liver cancer, and (iii) chronic gastritis and gastric cancer caused by *Helicobacter pylori* infection. Similarly, it has been postulated that having HT (as the most common type of thyroiditis) also carries an increased risk of TC. Many scholars have investigated this hypothesis, but most studies have been influenced by selection biases and imprecise indicators (11). Hence, studies have reached controversial (or even contrary) conclusions. Some studies have shown that HT may be a tumor-promoting factor (12). Other studies have reported HT to not have a relationship with the higher incidence of TC (13). HT has different influences on PTC in several aspects: certain clinical manifestations, sex, lymph-node metastasis, BRAF^V600E^ mutation, and recurrence (14–18). These factors affect the aggressiveness, treatment effect, and prognosis of PTC directly. Analyses of original studies and meta-analysis that had controversial conclusions revealed that biases usually arose because the (i) diagnostic criteria for HT were not uniform (B-ultrasound, antibodies, or pathology), (ii) the number of original research was limited, and (iii) the definition of the indicators used was not clear, such as lymph-node metastasis (metastasis of central lymph nodes or metastasis of lateral lymph nodes, or both).

Taking into account the limitations of such studies, an updated systematic review and meta-analysis is necessary. To better understand the impact of HT on PTC progression, we undertook a meta-analysis through a comprehensive search of the literature.

## 2 Methods

### 2.1 Registration

We registered (CRD42021265538) a systematic review entitled “The relationship between Hashimoto’s thyroiditis and thyroid carcinoma: a meta-analysis” in PROSPERO. We have been updating information in that systematic review.

### 2.2 Search Strategy

We followed the Meta-analysis of Observational Studies in Epidemiology (MOOSE) guidelines for carrying out and reporting this meta-analysis (19). The MOOSE checklist is shown in **Supplementary Table S1**. The literature from January 1, 2010 to December 31, 2020, regardless of region and publication type, was searched comprehensively in PubMed, Embase, Web of Science, and Cochrane Library databases. We used the following medical subject headings (MeSH) terms: “Thyroid neoplasms” and “Hashimoto disease”. We combined the MeSH terms and entry words when constructing searches. In addition, we also use the related article function to expand the search scope. If multiple original studies involving the same population were published, we selected the latest and most comprehensive research. Exported citations were managed, and duplicate data were deleted, using EndNote™ (https://endnote.com/).

### 2.3 Inclusion and Exclusion Criteria

The inclusion criteria were the following: (i) the study type must be prospective, retrospective, randomized controlled trial, or case–control; (ii) the research focus was HT and thyroid nodules, TC, or PTC; (iii) patients must be adults (≥18 years); and (iv) HT, TC, or PTC was diagnosed by pathology.

The exclusion criteria were the following: (i) the study design was not a prospective, retrospective, or randomized controlled trial; (ii) those that were editorials, letters, comments, case reports, or laboratory-animal research; and (iii) data were incomplete, and extracting the data needed in the meta-analysis was not possible.

According to these criteria, two researchers (JX and LM) conducted a preliminary screening by reading the title and abstract of the article. For the articles remaining after the preliminary screening, JX and LM read the full text and followed the inclusion and exclusion criteria strictly for final screening of the article. This screening process was carried out independently by JX and LM. Disputes in the screening process were resolved through negotiation or help of a third author (KD).

### 2.4 Extraction and Quality Assessment of Data

JX and LM used identical standardized data-extraction tables. The latter included the characteristics of the original research (author, publication year, region, and research type) and clinical characteristics of thyroid nodules or TC related to HT (main results were the malignancy prevalence of thyroid nodules and lymph-node metastasis in PTC; secondary results were multifocality, extrathyroidal expansion, BRAF^V600E^ mutation, recurrence, and distant metastasis). Disputes between JX and LM were resolved by KD. If the target data of the original study could not be obtained, then the corresponding author of the original study was contacted.

We used the Newcastle–Ottawa Scale (NOS) score to assess the quality of included studies (20). The NOS focuses on the choice of study subjects (four items), comparability between groups (two items) and measurement of results (three items, applicable to cohort studies), or exposure degree (three items, applicable to case–control studies). The NOS score of each study varied from 0 to 9 points (1 point for each item, 9 points in total). An original study with a NOS score ≥6 points was considered to be of “high quality.”

### 2.5 Statistical Analyses

The meta-analysis of all data was carried out using Revman 5.4 with Cochrane Training (https://training.cochrane.org/). The odds ratio (OR) and 95% confidence interval (95%CI) of each study were calculated. The χ^2^ test and I^2^ test were employed to evaluate heterogeneity. For the χ^2^ test, p < 0.10 was considered to denote significant heterogeneity. For the I^2^ test, heterogeneity types were divided into “none” (0%–25%), “mild” (25%–50%), “moderate” (50%–75%), and “severe” (75%–100%). If I^2^ > 50%, the random-effects model was chosen for the meta-analysis; otherwise, the fixed-effects model (FEM) was chosen. We drew funnel plots and observed their symmetry to identify publication bias, but this was mainly for meta-analysis containing ≥10 studies. If the number of included studies was too small, evaluation of the symmetry of the funnel chart was not possible. For sensitivity analysis, we observed changes in the overall results by eliminating individual studies one-by-one and utilizing different effect models.

## 3 Results

### 3.1 Literature Search

**Figure 1:**
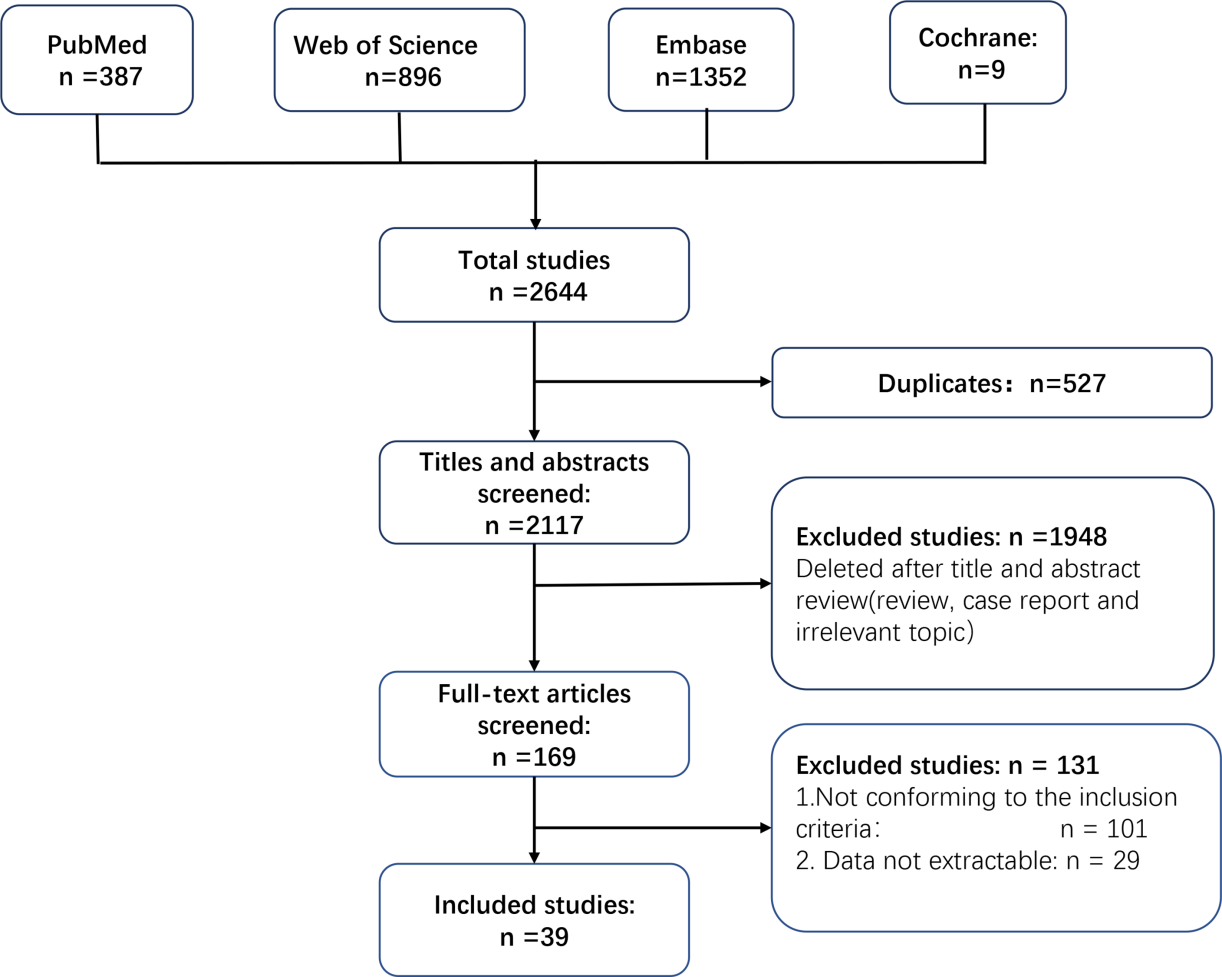
Flow diagram of systemic review procedure.

We identified 2,644 records in the last decade by searching PubMed, Embase, Web of Science, and Cochrane Library databases. A total of 527 studies were excluded because they contained duplicate data. A total of 1,948 studies were removed after screening of the title and abstract. JX and LM screened the full text of the remaining 169 studies independently. A total of 101 studies were excluded because they did not conform to the inclusion criteria, and an additional 29 studies were excluded because data could not be extracted. Finally, 39 studies were included for further analyses (14–16, 21–56). **Figure 1** is a flowchart of how studies were chosen.

### 3.2 Characteristics and Methodological Quality of Included Studies

The characteristics and methodological quality of included studies are described in detail in **Table 1**. The included studies were retrospective except for the studies by Cortes and colleagues (38) and Carvalho and collaborators (14). According to the NOS score, studies were of high quality except for the study by Kim and colleagues (26), which received a NOS score of 5 points.

**Table 1:** The clinical characteristics of included studies.

Author	Type of Study	Nation	Year of Publication	With HT/without HT	Pathological Type	NOS Quality Assessment Scale	Reference Number
Kim SS	Retrospective	Korea	2011	146/254	PTC	7	(21)
Kim HS	Retrospective	Korea	2010	105/218	PTMC(>5mm)	6	(22)
Huang BY	Retrospective	China	2011	85/1,703	PTC	7	(23)
Ahn D	Retrospective	Korea	2011	58/211	PTC	8	(24)
Jeong JS	Retrospective	Korea	2012	359/998	PTC	7	(25)
Kim YS	Retrospective	Korea	2013	316/931	PTC	5	(26)
Cordioli MI	Retrospective	Brazil	2013	35/59	PTC	8	(27)
Jara SM	Retrospective	America	2013	226/269	PTC	6	(28)
Marotta V	Retrospective	Italy	2013	54/92	PTC	6	(16)
Lim JY	Retrospective	Korea	2013	964/1,983	PTC	6	(29)
Lang BH	Retrospective	Korea	2014	331/514	PTC (<2cm)	6	(30)
Kwak HY	Retrospective	Korea	2014	40/306	PTC	7	(31)
Konturek A	Retrospective	Poland	2014	130/643	PTC	7	(32)
Kim SK	Retrospective	Korea	2015	1006/2,326	PTC	7	(33)
Kim SJ	Retrospective	Korea	2016	204/1,576	PTC	6	(34)
Carvalho MS	Prospective	Brazil	2016	191/442	PTC	7	(14)
Dobrinja C	Retrospective	Italy	2016	70/90	PTC	7	(35)
Zhang Y	Retrospective	China	2014	247/1,488	PTC	6	(36)
Girardi FM	Retrospective	Brazil	2015	148/269	PTC	6	(37)
Cortes MCS	Prospective	Brazil	2018	45/68	PTC	8	(38)
Lee I	Retrospective	Korea	2020	1,174/1,754	PTC	7	(15)
Yang Y	Retrospective	China	2014	92/199	PTMC	7	(39)
Zeng R-C	Retrospective	China	2016	222/397	PTC	8	(40)
Yoon YH	Retrospective	Korea	2012	56/139	PTC	7	(41)
Paulson LM	Retrospective	America	2012	61/78	PTC	8	(42)
Park JY	Retrospective	Korea	2014	169/484	PTC	7	(43)
Ding J	Retrospective	China	2020	233/1,106	PTC	8	(44)
Zhu F	Retrospective	China	2016	129/105	PTC	7	(45)
Song E	Retrospective	Korea	2018	305/1,064	PTC	8	(46)
Nam HY	Retrospective	Korea	2016	22/15	PTC	6	(47)
Giagourta I	Retrospective	Greece	2013	441/939	PTC	7	(48)
Consorti F	Retrospective	Italy	2010	24/76	PTC	6	(49)
Jackson D	Retrospective	America	2019	52/307	TC	6	(50)
Kim ES	Retrospective	Korea	2010	41/201	PTC	6	(51)
Lun Y	Retrospective	China	2013	256/2,222	PTC	6	(55)
Vasileiadis I	Retrospective	Greece	2014	246/590	PTC	6	(53)
Zhang L	Retrospective	China	2012	653/5,456	PTC	6	(54)
Paparodis R	Retrospective	America	2013	567/2,151	DTC	7	(55)
Zeng, Rong	Retrospective	China	2016	30/1,168	PTC	8	(56)

HT, Hashimoto’s thyroiditis; PTC, papillary thyroid carcinoma; PTMC, papillary thyroid microcarcinoma; TC, thyroid carcinoma; DTC, differentiated thyroid carcinoma.

In the included studies, most scholars focused on HT and its relationship to PTC. However, a few studies simultaneously analyzed multiple pathological types of TC (PTC, follicular, medullary, and undifferentiated).

### 3.3 HT as a Risk Factor of TC and PTC

**Figure 2:**
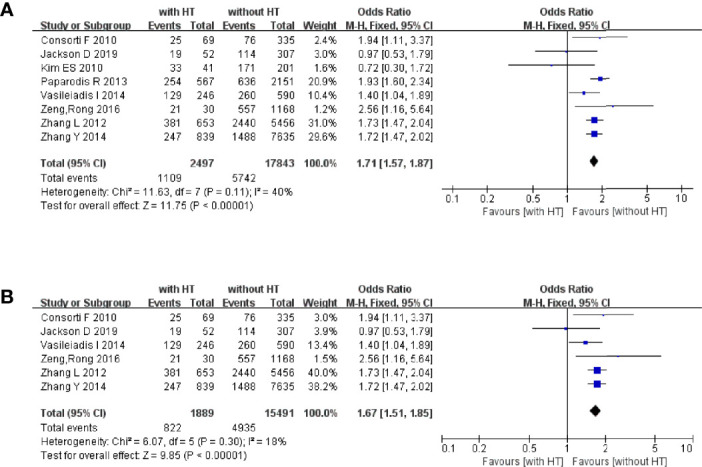
HT as a risk factor of TC and PTC: **(A)** forest plot of HT and TC and **(B)** forest plot of HT and PTC.

We wished to control for a confounding bias. Hence, we assessed if HT plays a part in multiple pathological types of TC (PTC, follicular, medullary, and undifferentiated) and whether HT is a risk factor in PTC patients (**Figure 2**).

In 2,497 HT patients, 1,109 were finally diagnosed with TC regardless of the pathological type (**Figure 2A**). In 17,847 patients without HT, 5,742 were diagnosed with TC. The pooled OR was 1.71 (95%CI, 1.57–1.80; p < 0.00001, I^2^ = 40%, FEM), which provided evidence that HT is a risk factor for TC.

Six studies focused on the relationship between HT and PTC (**Figure 2B**). In 1,889 HT patients, 822 were diagnosed with PTC. In 15,491 patients without HT, 4,935 were diagnosed with PTC. The pooled OR was 1.67 (95%CI 1.51–1.85; p < 0.00001, I^2^ = 18%, FEM).

### 3.4 HT and Multiple Clinical Characteristics in PTC Patients

#### 3.4.1 Multifocality and Extrathyroidal Extension

**Figure 3:**
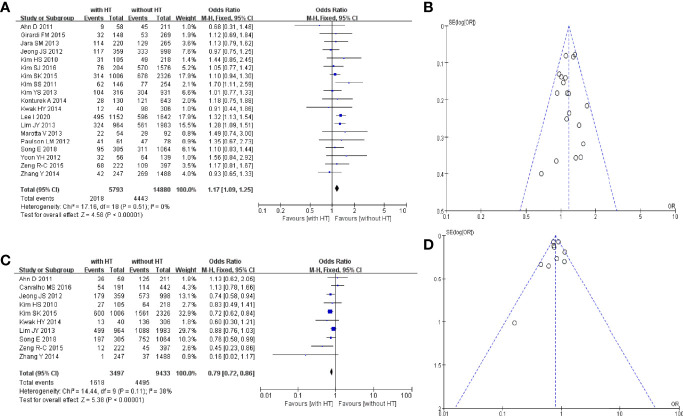
HT and multifocality of PTC patients: **(A)** forest plot and **(B)** funnel plot. HT and extrathyroidal extension of PTC patients: **(C)** forest plot and **(D)** funnel plot.

Nineteen studies reported the multifocality of PTC patients with or without HT. In PTC patients with HT, 2,018 of 5,793 patients had multifocal carcinomas. In PTC patients without HT, 4,443 of 14,880 patients had multifocal carcinomas (**Figure 3A**). The pooled OR was 1.17 (95%CI, 1.09–1.25; p < 0.00001, I^2^ = 0%, FEM). The funnel plot did not show an obvious publication bias (**Figure 3B**).

Ten studies reported the extrathyroidal extension of PTC patients with or without HT. In PTC patients with HT, 1,618 of 3,497 patients had an extrathyroidal extension. In PTC patients without HT, 4,495 of 9,433 patients had an extrathyroidal extension (**Figure 3C**). The pooled OR was 0.79 (95%CI, 0.72–0.86; p < 0.00001, I^2^ = 38%, FEM). The funnel plot did not show an obvious publication bias (**Figure 3D**).

#### 3.4.2 Lymph-Node Metastasis and Distant Metastasis

**Figure 4:**
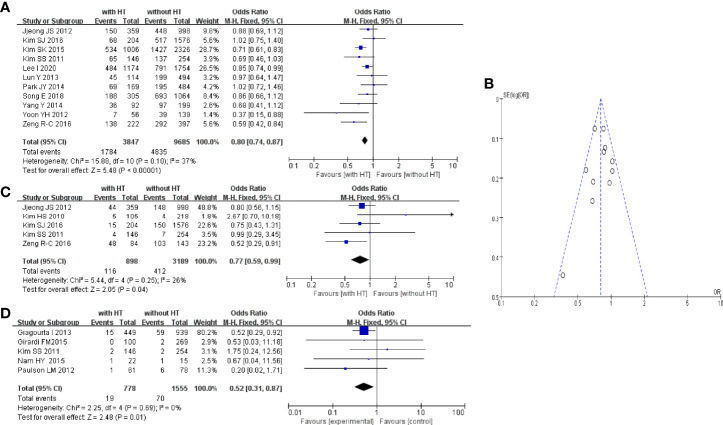
HT and central LN metastasis: **(A)** forest plot, **(B)** funnel plot; **(C)** Forest plot of HT and lateral LN metastasis; **(D)** forest plot of HT and distant metastasis.

Eleven studies reported metastasis of the central lymph nodes of PTC patients with or without HT. In PTC patients with HT, 1,784 of 3,847 patients had metastasis of the central lymph nodes. In PTC patients without HT, 4,835 of 9,685 patients had metastasis of the central lymph nodes (**Figure 4A**). The pooled OR was 0.80 (95%CI, 0.74–0.87; p < 0.00001, I^2^ = 37%, FEM). The funnel plot did not show an obvious publication bias (**Figure 4B**).

Five studies reported metastasis of lateral lymph nodes of PTC patients with or without HT. In PTC patients with HT, 116 of 898 patients had metastasis of lateral lymph nodes. In PTC patients without HT, 412 of 3,189 patients had metastasis of lateral lymph nodes (**Figure 4C**). The pooled OR was 0.77 (95%CI, 0.59–0.99; p = 0.04, I^2^ = 26%, FEM).

Five studies reported distant metastasis in PTC patients with or without HT. In PTC patients with HT, 19 of 778 patients had distant metastasis. In PTC patients without HT, 70 of 1,555 patients had distant metastasis (**Figure 4D**). The pooled OR was 0.52 (95%CI, 0.31–0.87; p = 0.01, I^2^ = 0%, FEM).

#### 3.4.3 BRAF^V600E^ Mutation and Recurrence

**Figure 5:**
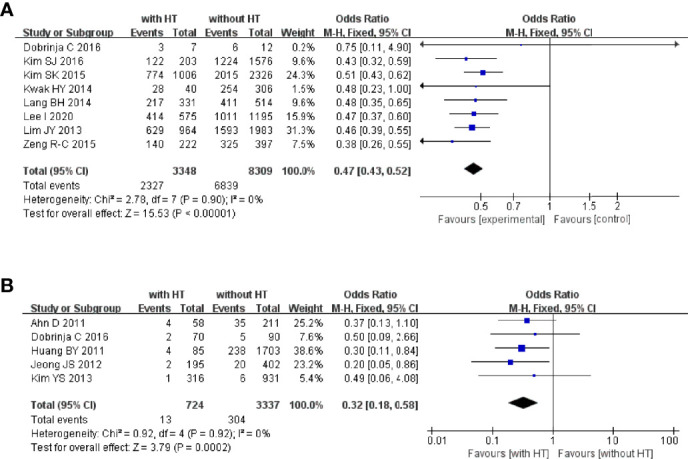
**(A)** Forest plot of HT and BRAFV600E mutation; **(B)** forest plot of HT and recurrence.

Eight studies reported the BRAF^V600E^ mutation in PTC patients with or without HT. In PTC patients with HT, 2,327 of 3,348 patients had the BRAF^V600E^ mutation. In PTC patients without HT, 6,839 of 8,309 patients had the BRAF^V600E^ (**Figure 5A**). The pooled OR was 0.47 (95%CI, 0.43–0.52; p < 0.00001, I^2^ = 0%, FEM).

Five studies reported the recurrence of PTC in patients with or without HT. In PTC patients with HT, 13 of 724 patients had tumor recurrence. In PTC patients without HT, 304 of 3,337 patients suffered tumor recurrence (**Figure 5B**). The pooled OR was 0.32 (95%CI, 0.18–0.58; p = 0.0002, I^2^ = 0%, FEM).

### 3.5 Sensitivity Analysis

Removal of the data of each study did not influence the pooled OR of the whole analysis. Transformation of the FEM or random-effects model did not influence the results. Hence, our analysis was reliable.

## 4 Discussion

The relationship between HT and TC was discussed first by Dailey in 1955 (57). Whether HT plays a part in the development and progression of TC has been controversial since then. We revealed a complicated relationship between HT and TC (especially PTC). HT appears to be a “double-edged sword” in TC. It is a potential risk factor in TC (especially PTC) patients. However, PTC patients with HT usually carry a better prognosis because they have a lower risk of extrathyroidal extension, BRAF^V600E^ mutation, metastasis, or recurrence.

The relationship between autoimmune thyroid disease and TC has been controversial for decades. Giagurta and colleagues compared the prevalence of autoimmune thyroiditis between people with PTC and individuals with benign thyroid nodules over the past 16 years. They found that patients with thyroid nodules and autoimmune thyroiditis were not more likely to have malignant thyroid nodules than individuals without autoimmune thyroiditis (48). Del Rio and colleagues conducted a prospective cohort study of 9,851 patients who underwent assessment of thyroid nodules between 1995 and 2017 (13). They showed that, for people with HT, the risk of malignant thyroid nodules was increased significantly. Our meta-analysis showed that the risk of TC or PTC in thyroid nodules for people with HT was increased. Therefore, we concluded that HT is a risk factor of TC or PTC.

Three possible pathogenic mechanisms can explain the results of our meta-analysis. First, the inflammatory response creates a favorable environment for malignant transformation. The damage wrought by cytokines and growth factors to stromal cells leads to changes in stromal reactivity, which, in turn, can lead to the malignant transformation of epithelial cells (58). In addition, the infiltration of immune cells to the thyroid gland may promote abnormal repair of DNA, thereby inducing PTC (59). Second, TSH is not only an endogenous stimulator of thyroid-hormone production, it is also a growth factor for thyroid cells (60). An increased level of TSH in most HT patients stimulates follicular epithelial hyperplasia, which promotes PTC. Third, expression of some oncogenes, such as RET/PTC gene rearrangement (61) and p63 mutation (62), may be involved in the transformation from HT to PTC. In contrast, the BRAF^V600E^ mutation, which is usually mutually exclusive with RET/PTC gene rearrangement (63), is more common in PTC without HT, a finding that is consistent with our meta-analysis.

Usually, TC (especially PTC) is considered to be less aggressive and to carry a better prognosis than that of a malignant tumor. However, the complicated extrathyroidal extension and various types of metastasis can be fatal in some circumstances. PTC shows a high tendency to spread to regional lymph nodes. The central region is the main region of lymph-node involvement in 20%–90% of PTC patients (64). Lymph-node metastasis is the main risk factor for PTC recurrence and is highly correlated with progression-free survival and overall survival in PTC patients (65, 66). Our meta-analysis showed PTC patients with HT to have a lower prevalence of lymph-node metastasis, distant metastasis, and recurrence. This conclusion is not consistent with the conclusions reached by Mao et al. (67) or Sun and colleagues (68). One reason for the different results could be a confounding bias (e.g., they did not distinguish lymph nodes from different areas).

According to our meta-analysis, PTC patients with HT had more favorable clinical characteristics and a better prognosis than PTC patients not suffering from HT. The latter is more common in young women. Thus, patients with HT are prone to be anxious and to undergo ultrasound of the thyroid gland frequently, so discovery of a malignant thyroid nodule at an early stage is likely. Multifocal carcinomas are independent risk factors for the TC prognosis. HT patients are more likely to have multifocal carcinomas, which leads to more aggressive and radical surgery, so their recurrence risk is lower than that of PTC patients without HT. Marotta and colleagues showed lymphocyte infiltration in HT patients to be a protective factor against PTC progression (16). Zhang et al. suggested that the BRAF^V600E^ mutation can help to predict the prognosis of PTC (69). The BRAF^V600E^ mutation is a marker of more aggressive behavior of PTC. In addition, the BRAF^V600E^ mutation is less common in PTC patients with HT than in PTC patients without HT. The results of our meta-analysis and the studies stated above suggest that HT is a protective factor against PTC progression, but the mechanism of action merits further study.

Our meta-analysis explored the relationship between PTC and HT from occurrence to progression, but had two main limitations. First, the included studies were mainly retrospective: more prospective studies and real-world studies are needed to draw more accurate conclusions. Second, the included studies involved mainly Asian patients. More prospective cohort studies involving multiple ethnicities are needed to further clarify the relationship between HT and PTC.

## 5 Conclusions

HT is a double-edged sword in TC patients. HT increases the risk of TC and PTC but is a protective factor against PTC progression.

## Data Availability Statement

The original contributions presented in the study are included in the article/**Supplementary Material**. Further inquiries can be directed to the corresponding author.

## Ethics Statement

The studies involving human participants were reviewed and approved by the ethics committee of Second Xiangya Hospital. The patients/participants provided their written informed consent to participate in this study. Written informed consent was obtained from the individual(s) for the publication of any potentially identifiable images or data included in this article.

## Author Contributions

JX and KD provided main effort in the procedure of meta-analysis and manuscript editing. LM, FY, and CG offered great help in the data extraction and data analysis. JH and CR gave many valuable suggestions to this article. CR also contributed to the manuscript revision. All authors contributed to the article and approved the submitted version.

## Funding

This work was supported by the National Key R&D Program of China under Grant 2019YFE0190500.

## Conflict of Interest

The authors declare that the research was conducted in the absence of any commercial or financial relationships that could be construed as a potential conflict of interest.

## Publisher’s Note

All claims expressed in this article are solely those of the authors and do not necessarily represent those of their affiliated organizations, or those of the publisher, the editors and the reviewers. Any product that may be evaluated in this article, or claim that may be made by its manufacturer, is not guaranteed or endorsed by the publisher.

## Abbreviations

HT, Hashimoto’s thyroiditis; TC, thyroid carcinoma; PTC, papillary thyroid carcinoma; TSH, thyroid-stimulating hormone
