# Supplementary material for: Hashimoto’s Thyroiditis: A “Double-Edged Sword” in Thyroid Carcinoma
Source: Front Endocrinol (Lausanne). 2022 Feb 24;13:801925. doi: 10.3389/fendo.2022.801925 (PMC8907134; doi:10.3389/fendo.2022.801925)
Supplement: Supplementary file 1 [file Table_1.pdf]

MOOSE Checklist for Meta-analyses of Observational Studies

Item No	Recommendation	Reported on Page No
Reporting of background should include		
1	Problem definition	2
2	Hypothesis statement	2
3	Description of study outcome(s)	2
4	Type of exposure or intervention used	2
5	Type of study designs used	7
6	Study population	4
Reporting of search strategy should include		
7	Qualifications of searchers (eg, librarians and investigators)	4-5
8	Search strategy, including time period included in the synthesis and key words	4
9	Effort to include all available studies, including contact with authors	4-5
10	Databases and registries searched	4
11	Search software used, name and version, including special features used (eg, explosion)	4
12	Use of hand searching (eg, reference lists of obtained articles)	4
13	List of citations located and those excluded, including justification	Figure 1
14	Method of addressing articles published in languages other than English	NA
15	Method of handling abstracts and unpublished studies	5
16	Description of any contact with authors	5
Reporting of methods should include		
17	Description of relevance or appropriateness of studies assembled for assessing the hypothesis to be tested	5
18	Rationale for the selection and coding of data (eg, sound clinical principles or convenience)	5
19	Documentation of how data were classified and coded (eg, multiple raters, blinding and interrater reliability)	5-6
20	Assessment of confounding (eg, comparability of cases and controls in studies where appropriate)	7
21	Assessment of study quality, including blinding of quality assessors, stratification or regression on possible predictors of study results	Table 1
22	Assessment of heterogeneity	6-9
23	Description of statistical methods (eg, complete description of fixed or random effects models, justification of whether the chosen models account for predictors of study results, dose-response models, or cumulative meta-analysis) in sufficient detail to be replicated	6
24	Provision of appropriate tables and graphics	Table 1 & Figure 2-5
Reporting of results should include		
25	Graphic summarizing individual study estimates and overall estimate	Figure 2-5
26	Table giving descriptive information for each study included	Table 1
27	Results of sensitivity testing (eg, subgroup analysis)	9
28	Indication of statistical uncertainty of findings	6-9

Item No	Recommendation	Reported on Page No
Reporting of discussion should include		
29	Quantitative assessment of bias (eg, publication bias)	11-12
30	Justification for exclusion (eg, exclusion of non-English language citations)	11-12
31	Assessment of quality of included studies	Table 1
Reporting of conclusions should include		
32	Consideration of alternative explanations for observed results	12
33	Generalization of the conclusions (ie, appropriate for the data presented and within the domain of the literature review)	12
34	Guidelines for future research	12
35	Disclosure of funding source	12

From: Stroup DF, Berlin JA, Morton SC, et al, for the Meta-analysis Of Observational Studies in Epidemiology (MOOSE) Group. Meta-analysis of Observational Studies in Epidemiology. A Proposal for Reporting. *JAMA*. 2000;283(15):2008-2012. doi: 10.1001/jama.283.15.2008.

Transcribed from the original paper within the NEUROSURGERY® Editorial Office, Atlanta, GA, United Sates. August 2012.
